# Social attention in anorexia nervosa and autism spectrum disorder: Role of social motivation

**DOI:** 10.1177/13623613211060593

**Published:** 2021-11-30

**Authors:** Jess Kerr-Gaffney, Emily Jones, Luke Mason, Hannah Hayward, Declan Murphy, Eva Loth, Kate Tchanturia

**Affiliations:** 1 King’s College London, UK; 2 University of London, UK; 3 South London and Maudsley NHS Trust, UK; 4 Ilia State University, Georgia

**Keywords:** anorexia nervosa, autism spectrum disorder, eye-tracking, social attention, social motivation

## Abstract

Research suggests a relationship between anorexia nervosa and autism spectrum disorder. The aim of the current study was to examine social attention in anorexia nervosa and autism spectrum disorder compared with age- and sex-matched typically developing groups, and to examine whether lowered social motivation could explain reductions in social attention across the two disorders. Participants’ eye movements were tracked while watching a dynamic social scene. The proportion of fixation duration to faces, bodies and non-social areas of interest were compared across groups. Participants with autism spectrum disorder looked at faces significantly less often than controls, however, there were no differences between anorexia nervosa and controls in attention to faces. Typically developing -normed *z*-scores indicated that attention to faces showed the greatest deviation from normative data compared with body or non-social areas of interest in both autism spectrum disorder and anorexia nervosa, however, differences were larger in autism spectrum disorder than in anorexia nervosa. Social motivation scores did not predict attention to faces in either autism spectrum disorder or anorexia nervosa. Our results do not support the hypothesis that differences in social motivation underlie reduced social attention in both anorexia nervosa and autism spectrum disorder.

**Lay abstract:**

Research suggests a relationship between autism and anorexia nervosa. For example, rigid and inflexible behaviour, a preference for routine and social difficulties are seen in both conditions. In this study, we examined whether people with anorexia and people with autism show similarities in social attention (where they look while engaging in social interactions or watching a scene with people interacting). This could help us understand why people with anorexia and autism experience difficulties in social situations. Participants with either anorexia or autism, as well as participants with no mental health problems watched a video of a social scene while we recorded which parts of the scene they looked at with an eye-tracker. Participants also completed questionnaires to assess characteristics of autism. We found that autistic participants looked at faces less than typically developing participants. However, participants with anorexia did not show a similar reduction in attention to faces, contrary to our predictions. Autistic features were not related to attention in either group. The results suggest that autistic people may miss important social cues (like facial expressions), potentially contributing to social difficulties. However, this mechanism does not appear explain social difficulties in people with anorexia.

## Introduction

Anorexia nervosa (AN) is a severe psychiatric disorder characterised by persistent behaviour to restrict energy intake, intense fears of weight gain and a disturbance in the way one’s body weight or shape is experienced (American Psychiatric Association [APA], 2013). AN usually emerges in adolescence, with a peak age of onset between 15 and 19 years (Herpertz-Dahlmann et al., 2015). Mostly females are affected, with epidemiological studies reporting male to female sex ratios of around 1:4 (Bulik et al., 2006). A range of temperamental, social and biological factors are thought to contribute to the development and maintenance of AN; however, no single psychological or pharmacological intervention has proven to be particularly effective in treating the disorder. AN has a significant impact on psychosocial functioning and mental health. For example, individuals with AN show high levels of social disability, similar to those with schizophrenia (Rymaszewska et al., 2007) and obsessive compulsive disorder (OCD) (Tchanturia et al., 2013). Psychiatric comorbidity is also common; around 70% of individuals with AN have at least one additional Axis 1 disorder (Ulfvebrand et al., 2015). Research has mostly focused on depressive and anxiety disorders, and suggests that the presence of these comorbidities is associated with poorer outcomes in AN (Franko et al., 2018; Yackobovitch-Gavan et al., 2009; Zerwas et al., 2013).

Recently, research has accumulated to suggest a relationship between AN and autism spectrum disorder (ASD) (Kinnaird & Tchanturia, 2021). ASD is a neurodevelopmental disorder characterised by difficulties in social communication and interaction, as well as restricted and repetitive behaviours or interests (APA, 2013). ASD affects around 1% of the population, with a male to female sex ratio of around 3:1 (Brugha et al., 2011; Loomes et al., 2017). A proportion of those with AN show high levels of ASD features, with around one-third scoring above the clinical cut-off on clinical interview measures such as the Autism Diagnostic Observation Schedule, 2nd edition (ADOS-2; Westwood & Tchanturia, 2017). These features are also present in those recovered from AN, suggesting that ASD symptoms may not be side effects of starvation (Bentz et al., 2017; Kerr-Gaffney, Hayward, et al., 2021). However, some studies suggest improvements in social functioning and cognitive flexibility after recovery from AN (Kucharska et al., 2019; Oldershaw et al., 2010). Similarly, individuals with ASD show significantly more eating disorder symptoms than non-autistic people, with around 27% of women with ASD reporting clinically significant levels of eating disorder symptoms (Kalyva, 2009; Nickel et al., 2019; Spek et al., 2020).

Furthermore, similarities in both neuropsychological and socio-emotional functioning have been documented in individuals with AN and those with ASD. For example, difficulties in set-shifting, weak central coherence and superior attention to detail are apparent in both disorders (Happé & Booth, 2008; Jolliffe & Baron-Cohen, 1997; Lang, Lopez, et al., 2014; Lang, Stahl, et al., 2014; Shah & Frith, 1993; Westwood, Stahl et al., 2016). Difficulties in theory of mind (ToM) are also associated with AN and ASD (Bora & Kose, 2016; Frith & Happé, 1994). These characteristics are seen in the acute stage of AN, with mixed evidence for persistence after recovery (Danner et al., 2012; Harrison et al., 2010; Oldershaw et al., 2010). Similar findings have been demonstrated in first-degree relatives of those with AN (Holliday et al., 2005; Kanakam et al., 2013; Roberts et al., 2010; Tenconi et al., 2010) and those with ASD (Bölte & Poustka, 2006; Eyuboglu et al., 2018; Happe et al., 2003; Nagar Shimoni et al., 2012; Wong et al., 2006), suggesting that these characteristics may be heritable trait markers for both disorders. In this way, similarities in neuro- or socio-cognitive functioning and behavioural expression (e.g. rigidity and adherence to routines) are hypothesised to be caused by similar genetic variants in ASD and AN (Zhou et al., 2018). Regarding ToM specifically, a recent meta-analysis demonstrated that people with AN and those with ASD show difficulties in all aspects of ToM compared with typically developing (TD) participants; however, these differences were generally greater in ASD than in AN (Leppanen et al., 2018). These differences between AN and ASD in the magnitude of ToM difficulties may reflect a paucity of research in AN (as few as five studies were found for some ToM domains), or may suggest differences in the underlying processes responsible for social understanding in AN and ASD.

Attending to others’ eye gaze and facial expression is an important precursor to social understanding, as these non-verbal cues convey important information about an individual’s emotions and intentions. In typical development, social information is highly salient, with infants as young as a few days old showing a preference for faces (Reynolds & Roth, 2018). However, reductions in social attention are among the earliest symptoms of ASD (Jones et al., 2014). On average, individuals with ASD pay less attention to faces and eyes, and show increased attention to non-social aspects of a scene compared with TD (Frazier et al., 2017). Some evidence also suggests that reductions in attention to faces predict the degree of social impairment and emotion recognition difficulties in people with ASD (Corden et al., 2008; Falkmer et al., 2011; Müller et al., 2016). Accordingly, a small number of studies have begun to investigate social attention in individuals with AN, finding reductions in attention to faces (Kerr-Gaffney, Mason, et al., 2021; Watson et al., 2010) and eyes (Harrison et al., 2019) compared with the controls. One of these studies demonstrated that reductions in attention to faces in AN were fully mediated by self-reported ASD symptoms (Kerr-Gaffney, Mason, et al., 2021). Although this finding suggests that reduced attention to faces in AN is a result of the high levels of ASD symptoms within this population, the exact way in which ASD symptoms may influence social attention in AN is not yet known.

One possible explanation for reductions in social attention in both AN and ASD is low social motivation. Social motivation encompasses several psychological dispositions that bias humans to attend to social stimuli, seek and take pleasure from social interactions, and maintain relationships. It is hypothesised that low social motivation is a primary characteristic of ASD, which causes downstream effects on the development of social cognition (Chevallier et al., 2012). Evidence suggests that individuals with ASD are on average less responsiveness to social rewards (Demurie et al., 2011), and experience higher levels of social anhedonia (a lack of pleasure from social interaction) than TD (Chevallier, Grèzes, et al., 2012). Furthermore, adults with ASD have fewer friendships than those without ASD (Baron-Cohen & Wheelwright, 2003; Howlin et al., 2004), are less likely to initiate social cues (Liebal et al., 2008), and use fewer strategies to preserve their reputation and self-image (Barbaro & Dissanayake, 2007; Chevallier, Molesworth, et al., 2012; Izuma et al., 2011). Similarly, individuals with AN report having fewer friends and engaging in more solitary activities than TD both before and during the illness (Adenzato et al., 2012; Cardi et al., 2018; Fairburn et al., 1999; Krug et al., 2012; Westwood, Lawrence et al., 2016). They also report higher levels of social anhedonia (Harrison et al., 2014; Tchanturia et al., 2012), and show lower responsiveness to social reward compared with TD (Watson et al., 2010). Low levels of social motivation persist after recovery from AN, similar to those reported in the acute state and to those reported by females with ASD (Kerr-Gaffney, Hayward, et al., 2021). Together, these results suggest that social motivation may be altered in both AN and ASD, possibly representing a heritable trait conferring increased risk to both disorders (Jones et al., 2017; Sung et al., 2005; Uljarević et al., 2021).

Thus far, no studies have directly compared social attention in participants with ASD and participants with AN, nor have they investigated social motivation as a possible transdiagnostic factor relating to attentional differences in these populations. Thus, the aim of the current study was to compare social attention in AN and ASD to age- and sex-matched TD groups, while viewing a naturalistic, dynamic social scene. We also compared the AN and ASD groups directly using age and sex-adjusted social attention *z*-scores (representing the degree of difference between each individual with AN or ASD and their respective control group). Furthermore, we aimed to examine which dimensions of autistic symptoms (measured by the Social Responsiveness Scale (SRS-2)) contribute to differences in social attention across AN and ASD. We hypothesised that both participants with AN and those with ASD would show reduced attention to faces compared with TDs. Regarding *z*-scores, we hypothesised that attention to faces in participants with AN and those with ASD be more atypical (i.e. deviating further from that of the TD sample) than attention to non-social aspects of the scene. Finally, we hypothesised that higher scores on the social motivation subscale of the SRS-2 (reflecting more atypicality) would predict reduced attention to faces in both AN and ASD.

## Method

### Participants

Four groups of participants were included in the study: AN, ASD and two sex- and age-matched typically developing control groups (TD-AN and TD-ASD). Data were extracted from two existing data sets: AN and TD-AN were from a study investigating social and emotional functioning in AN, while participants with ASD and TD-ASD were from the European Autism Interventions Longitudinal European Autism Project (EU-AIMS LEAP) (Loth et al., 2017). Details regarding participant recruitment for the original studies can be found in Supplementary File 1. The study received ethical approval from the National Health Service Research Ethics Committee (Camberwell St Giles, 17/LO/1960).

The following inclusion criteria were applied to all participants: aged ⩾18 years, and average or above average IQ (⩾85). In the original AN study, the upper age limit for AN and TD-AN participants was 55 years, while in the EU-AIMS LEAP study, the upper age limit for participants with ASD and TD-ASD was 30 years. Additional inclusion criteria for participants with AN were a BMI ⩽ 18.5, and fulfilling criteria for current AN according to the Structured Clinical Interview for *Diagnostic and Statistical Manual of Mental Disorders*, 5th edition (*DSM*-5), research version (SCID-5-RV) (First et al., 2015). TD groups were screened using the SCID-5-RV to ensure that they did not show symptoms consistent with any psychiatric disorders, were not using psychiatric medication at the time of testing, and had a BMI between 18.5 and 25. Participants with ASD met *Diagnostic and Statistical Manual of Mental Disorders*, 4th edition (*DSM*-4) (APA, 2000), *DSM*-5 (APA, 2013) or International Classification of Diseases (ICD)-10 (World Health Organization, 1993) criteria. ASD diagnoses were based on a comprehensive assessment of the participant’s clinical history and current symptoms prior to enrolment in the original study.

No participants with AN had an existing diagnosis of ASD. However, information regarding possible comorbid eating disorder diagnoses was not available for participants with ASD.

### Measures

The eye-tracking stimulus material was a clip from the 1995 film, *Welcome to the Dollhouse*. The clip is 124-s long and depicts a naturalistic social situation in which a young female is attempting to find a place to sit in a school cafeteria. The stimulus has previously shown sensitivity to differences in attention in children with ASD compared with TD (Rice et al., 2012). Participants were asked to view the clip as they would watch television. After interpolating periods of missing data of 200 ms or shorter and bracketed by looking at the same area of interest (AOI) (to account for blinks), total looking times (in seconds) to the screen were computed to control for overall attention to the stimulus, and total fixation duration to each AOI was calculated (as a proportion of total valid samples).

**Figure 1.**
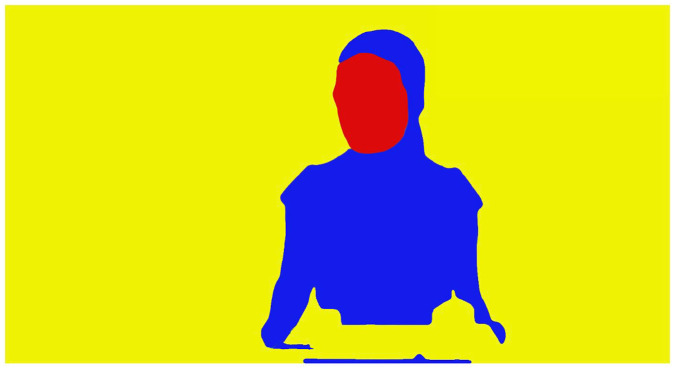
A single frame from the video stimulus with the areas of interest (AOIs) overlaid. Faces are highlighted in red, bodies in blue and non-social regions in yellow.

Figure 1 depicts a frame from the video with the AOIs overlaid. AOIs were drawn on each individual frame of the video using Apple Motion (Apple Inc., 2019). To capture social attention, we measured fixation duration to the face AOI, as well as fixation duration to non-social background regions. As body stimuli may be salient to individuals with AN (Pinhas et al., 2014), we also examined the proportion of looking time to the body AOI.

The SRS-2 (Constantino & Gruber, 2012) is a self-report questionnaire measuring characteristics associated with ASD. Higher scores indicate more severe symptoms. It has five subscales: social awareness, social cognition, social communication, social motivation, and restricted interests and repetitive behaviours. T scores for both total and subscales were used in this study, as these are standardised by age and sex. T scores are interpreted as follows: ⩽59 within normal limits, 60–65 mild symptoms, 66–75 moderate symptoms and ⩾76 severe symptoms.

The eating disorder examination questionnaire (EDE-Q; Fairburn & Beglin, 1994) is a self-report questionnaire assessing severity of eating disorder psychopathology. The total scores are calculated by averaging responses across all items. Higher scores indicate more severe symptoms (max. score 6). The EDE-Q was collected in AN and TD-AN only.

The Hospital Anxiety and Depression Scale (HADS; Zigmond & Snaith, 1983) measured anxiety and depression in AN and TD-AN, while the Beck Anxiety and Depression Inventories (Beck & Steer, 1993; Beck et al., 1996) measured anxiety and depression in participants with ASD and TD-ASD. Higher scores on these self-report questionnaires indicate more severe psychopathology. As different measures were used across studies, *z*-scores were calculated. For participants with AN, *z*-scores were based on the mean and standard deviation (*SD*) of the TD-AN group. For participants with ASD, *z*-scores were based on the mean and *SD* of TD-ASD.

IQ was measured using the Wechsler Abbreviated Scales of Intelligence-Second Edition (WASI-II) (Wechsler, 2011) in all participants with AN and TD-AN, and 44.9% of participants with ASD and TD-ASD. The WASI-I (1.3%), WAIS-*R* (19.6%), WAIS-III (15.2%) and WAIS-IV (19.0%) were used to measure IQ in the remainder of participants with ASD and TD-ASD.

Height and weight were measured at the study session to calculate BMI (weight/height^2^).

### Procedure

Participants viewed the video clip while their eye movements were recorded using a Tobii TX300 eye-tracker. The desktop mounted eye-tracker had a sampling rate of 300 Hz, a screen resolution of 1,920 × 1,080, and a diagonal screen size of 23 in. During tracking, infrared diodes generate reflections on the participant’s retinas and corneas. From this reflection, the angular rotation of each eye is estimated. Before stimulus presentation, a five-point calibration procedure was run. Calibration relates the angular rotation of each eye to the corresponding *x* and *y* coordinates on the screen surface. Participants were seated approximately 60 cm from the screen. Stimulus presentation, behavioural data and eye-tracking data were managed and recorded using custom-written MATLAB software (Task Engine, 2015).

Participants completed questionnaires either online prior to the eye-tracking session or with the researcher afterwards.

### Analysis

Histograms and Q–Q plots were inspected to check for normal distributions. Group differences in demographic variables and clinical characteristics between the clinical groups (AN and ASD) and their respective TD groups were assessed using independent samples *t* tests, or Mann–Whitney U tests if data were non-normally distributed. Chi-square tests were conducted for dichotomous variables (or Fisher’s exact test where the sample size assumption was not met). Mixed ANOVAs with the between-subjects factor group (AN and TD-AN; ASD and TD-ASD) and the within-subjects factor AOI (face, body, non-social) were run to compare patterns of attention in the clinical groups with their respective control groups. To compare attention to face, body and non-social AOIs in participants with AN and participants with ASD, we used a normative modelling approach (Marquand et al., 2019) to compute *z*-scores relative to the age and sex-modelled data from the respective TD groups. Specifically, statistical models were estimated to model variance in social attention from sex and age across the reference cohorts using Gaussian process regression. These models were then used to quantify the deviations of individual samples from the ASD and AN cohorts with respect to the reference models. *z*-scores in AN and ASD were then compared using a mixed ANOVA with the between-subjects factor group (AN and ASD) and the within-subjects factor AOI (face, body, non-social). Effect sizes are reported for *t* tests (Cohen’s *d*), Chi-square tests (odds ratio (OR)), Mann–Whitney U tests (η^2^) and mixed ANOVAs (η_*p*_^2^).

To examine the associations between social attention and ASD features, Pearson’s correlations were run between the proportion of time spent looking at the face, body and non-social AOIs, and each subscale of the SRS-2. Anxiety and depression scores were also included in correlation analyses, as these symptoms could conceivably also be related to social attention. Where significant correlations were found, variables were entered into linear regressions to determine whether ASD features and/or psychopathology predicted attention to AOIs. A significance level of α = 0.01 was used across analyses; however, no correction was applied to account for multiple comparisons (Green & Britten, 1998).

### Community involvement

There was autistic representation within the research team.

## Results

### Demographic characteristics

In total, there were 260 participants eligible for inclusion. Eighteen participants were subsequently excluded due to low quality eye-tracking data, defined as a proportion of valid samples of less than 0.25. Thus, data from 242 participants were included in analyses (43 AN, 41 TD-AN, 93 ASD, 65 TD-ASD). Demographic information and psychopathology scores across groups are displayed in Table 1. Participants with AN had a mean illness duration of 7.22 years (*SD* = 8.06); 51.2% of participants with AN and 29.1% of participants with ASD were taking psychiatric medication at the time of testing.

**Table 1.** Mean (*SD*) demographic characteristics, autistic symptoms and psychopathology scores.

	AN (*n* = 43)	TD-AN (*n* = 41)	ASD (*n* = 93)	TD-ASD (*n* = 65)	AN vs TD-AN test statistics	ASD vs TD-ASD test statistics	AN vs ASD test statistics
Age (years)	25.27 (5.49)	24.57 (4.57)	22.88 (3.60)	23.77 (2.96)	*p* = 0.541, 99% CI: –3.69, 2.30, *d* = 0.14	*p* = 0.090, 99% CI: –0.47, 2.26, *d* = 0.27	*p* = 0.017, 99% CI: –0.20, 4.97, *d* = 0.51
Sex (female %)	90.7	95.1	29.0	29.2	*p* = 0.676, 99% CI: 0.05, 5.02, OR = 0.50	*p* = 0.978, 99% CI: 0.40, 2.48, OR = 0.99	***p*** **< 0.001**, 99% CI: 5.45, 104.16, OR = 23.83
IQ	110.45 (12.41)	112.38 (7.44)	108.54 (12.76)	110.93 (12.08)	*p* = 0.395, 99% CI: −4.04, 7.88, *d* = 0.19	*p* = 0.238, 99% CI: −2.86, 7.65, *d* = 0.19	*p* = 0.418, 99% CI: −4.24, 8.06, *d* = 0.15
BMI	15.71 (1.42)	21.67 (1.94)	23.36 (5.05)	22.38 (1.80)	***p*** **< 0.001**, 99% CI: 4.98, 6.93, *d* = 3.51	*p* = 0.089, 99% CI: −2.48, 0.52, *d* = 0.26	***p*** **< 0.001**, 99% CI: −9.14, −6.16, *d* = 2.06
Anxiety *z*-score	2.43 (1.35)	0.00 (1.00)	3.51 (3.92)	0.00 (1.00)	***p*** **< 0.001**, 99% CI: −3.12, −1.75, *d* = 2.05	***p*** **< 0.001**, 99% CI: −4.74, −2.26, *d* = 1.23	*p* = 0.031, 99% CI: −2.38, 0.22, *d* = 0.37
Depression *z*-score	3.92 (2.17)	0.00 (1.00)	3.09 (3.57)	0.00 (1.00)	***p*** **< 0.001**, 99% CI: −4.89, −2.95, *d* = 2.32	***p*** **< 0.001**, 99% CI: −4.23, −1.94, *d* = 1.18	*p* = 0.116, 99% CI: −0.55, 2.21, *d* = 0.28
EDE-Q total^a^	3.97 (1.92)	0.35 (0.86)	–	–	***p*** **< 0.001**, η^2^ = 0.68	–	–
SRS-2 *t* scores							
Total	62.60 (11.58)	48.62 (8.91)	65.38 (8.48)	53.33 (4.12)	***p*** **< 0.001**, 99% CI: −20.07, −7.89, *d* = 1.35	***p*** **< 0.001**, 99% CI: −14.97, −9.12, *d* = 1.81	*p* = 0.176, 99% CI: −8.17, 2.61. *d* = 0.27
Social awareness	50.24 (9.41)	45.44 (8.33)	61.19 (7.44)	59.54 (6.44)	*p* = 0.018, 99% CI: −10.03, −0.42, *d* = 0.54	*p* = 0.187, 99% CI: −4.93, 1.61, *d* = 0.24	***p*** **< 0.001**, 99% CI: −15.07, −6.85, *d* = 1.29
Social cognition	60.21 (11.83)	48.46 (9.61)	66.58 (8.24)	57.39 (5.38)	***p*** **< 0.001**, 99% CI: −18.10, −5.40, *d* = 1.09	***p*** **< 0.001**, 99% CI: −12.31, −6.08, *d* = 1.32	***p*** **= 0.003**, 99% CI: −11.82, −0.92, *d* = 0.62
Social communication	58.83 (11.29)	47.31 (9.40)	65.08 (8.92)	53.07 (4.30)	***p*** **< 0.001**, 99% CI: −18.64, −6.41, *d* = 1.11	***p*** **< 0.001**, 99% CI: −15.08, −8.93, *d* = 1.72	***p*** **= 0.006**, 99% CI: −10.18, −0.31, *d* = 0.61
Social motivation	67.81 (11.92)	53.08 (9.29)	61.49 (7.35)	52.03 (4.10)	***p*** **< 0.001**, 99% CI: −20.98, −8.48, *d* = 1.38	***p*** **< 0.001**, 99% CI: −12.09, −6.82, *d* = 1.59	***p*** **= 0.003**, 99% CI: 0.93, 11.70, *d* = 0.64
Restricted interests and repetitive behaviours	65.33 (12.94)	49.62 (7.78)	64.01 (11.98)	46.39 (5.26)	***p*** **< 0.001**, 99% CI: −21.95, −9.48, *d* = 1.47	***p*** **< 0.001**, 99% CI: −21.66, −13.58, *d* = 1.90	*p* = 0.578, 99% CI: −4.87, 7.51, *d* = 0.11

AN: anorexia nervosa; ASD: autism spectrum disorder; BMI: body mass index; CI: confidence intervals; EDE-Q: eating disorder examination questionnaire; IQ: intelligence quotient; OR: odds ratio; *SD*: standard deviation; SRS-2: social responsiveness scale, 2nd edition; TD-AN: typically developing, matched to anorexia nervosa group; TD-ASD: typically developing, matched to autism spectrum disorder group.

a Median and interquartile range (non-normally distributed data).

Significant differences (*p* < 0.01) between AN and ASD and their control groups are highlighted in bold.

Regarding ASD features, both AN and ASD groups showed significantly higher total SRS-2 scores compared with their respective control groups, with large effect sizes. Total scores in the clinical groups did not differ from one another, and were within the mild symptom range, indicating clinically significant difficulties in social behaviour, leading to mild to moderate interference with everyday life. Both participants with AN and participants with ASD scored significantly higher than their respective control groups on all SRS-2 subscales apart from the social awareness subscale. Participants with ASD scored significantly higher than AN on the social awareness, social cognition and social communication subscales, but did not differ on the restricted interests and repetitive behaviour subscale. Finally, participants with AN scored significantly higher than ASD on the social motivation subscale.

### Social attention

**Figure 2.**
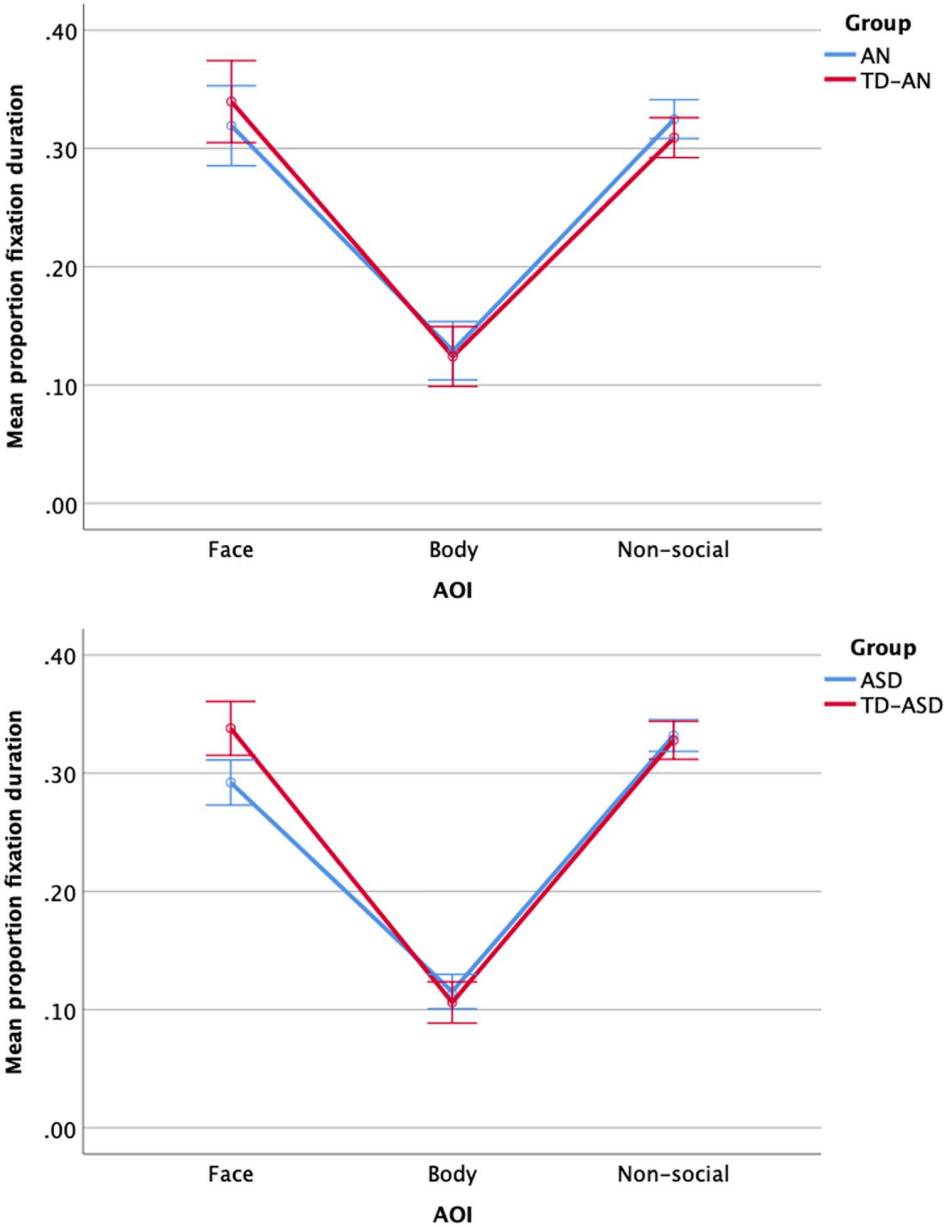
Mean proportion of time spent looking at body, face and non-social areas of interest (AOIs) in AN versus TD-AN (top) and ASD versus TD-ASD (bottom). Error bars represent 99% confidence intervals.

The clinical groups (AN and ASD) were compared with their respective control groups in the proportion of time spent looking at each AOI (face, body, non-social) with mixed ANOVAs, displayed in Figure 2. For AN and TD-AN comparisons, Mauchley’s test of sphericity was significant (*p* < 0.001), therefore a Greenhouse–Geisser correction was used. There was no significant interaction between AOI and group, *F*(1.59, 130.60) = 1.24, *p* = 0.286, η_*p*_^2^ = 0.02. The main effect of AOI was significant, *F*(1.59, 130.60) = 188.38, *p* < 0.001, η_*p*_^2^ = 0.70. Participants looked at faces (AN *M* = 0.32, *SD* = 0.09; TD-AN *M* = 0.34, *SD* = 0.08) and non-social aspects of the scene (AN *M* = 0.32, *SD* = 0.04; TD-AN *M* = 0.31, *SD* = 0.04) more than bodies (AN *M* = 0.13, *SD* = 0.07; TD-AN *M* = 0.12, *SD* = 0.05), both *p* < 0.001. There was no significant main effect of group, *F*(1,82) = 0.00, *p* = 0.987, η_*p*_^2^ = 0.00.

For ASD and TD-ASD comparisons, Mauchley’s test of sphericity was significant (*p* < 0.001), therefore a Greenhouse–Geisser correction was used. There was a significant interaction between AOI and group, *F*(1.79, 279.70) = 7.33, *p* = 0.001, η_*p*_^2^ = 0.05. Participants with ASD (*M* = 0.29, *SD* = 0.07) looked at the face AOI significantly less than TD-ASD (*M* = 0.34, *SD* = 0.07), *p* < 0.001. There were no significant group differences in attention to the body AOI (ASD *M* = 0.12, *SD* = 0.06, TD-ASD *M* = 0.11, *SD* = 0.05), *p* = 0.294 or non-social AOI (ASD *M* = 0.33, *SD* = 0.04, TD-ASD *M* = 0.33, *SD* = 0.06), *p* = 0.616. There was a significant effect of AOI on looking times in ASD, *F*(1.70, 156.48) = 266.63, *p* < 0.001, η_*p*_^2^ = 0.74. Participants looked at non-social AOIs more than faces and bodies (both *p* < 0.001), and faces more than bodies (*p* < 0.001). There was also a significant effect of AOI on looking times in TD-ASD, *F*(1.65, 105.54) = 219.90, *p* < 0.001, η_*p*_^2^ = 0.78; participants looked at faces and non-social AOIs more than bodies (both *p* < 0.001).

**Figure 3.**
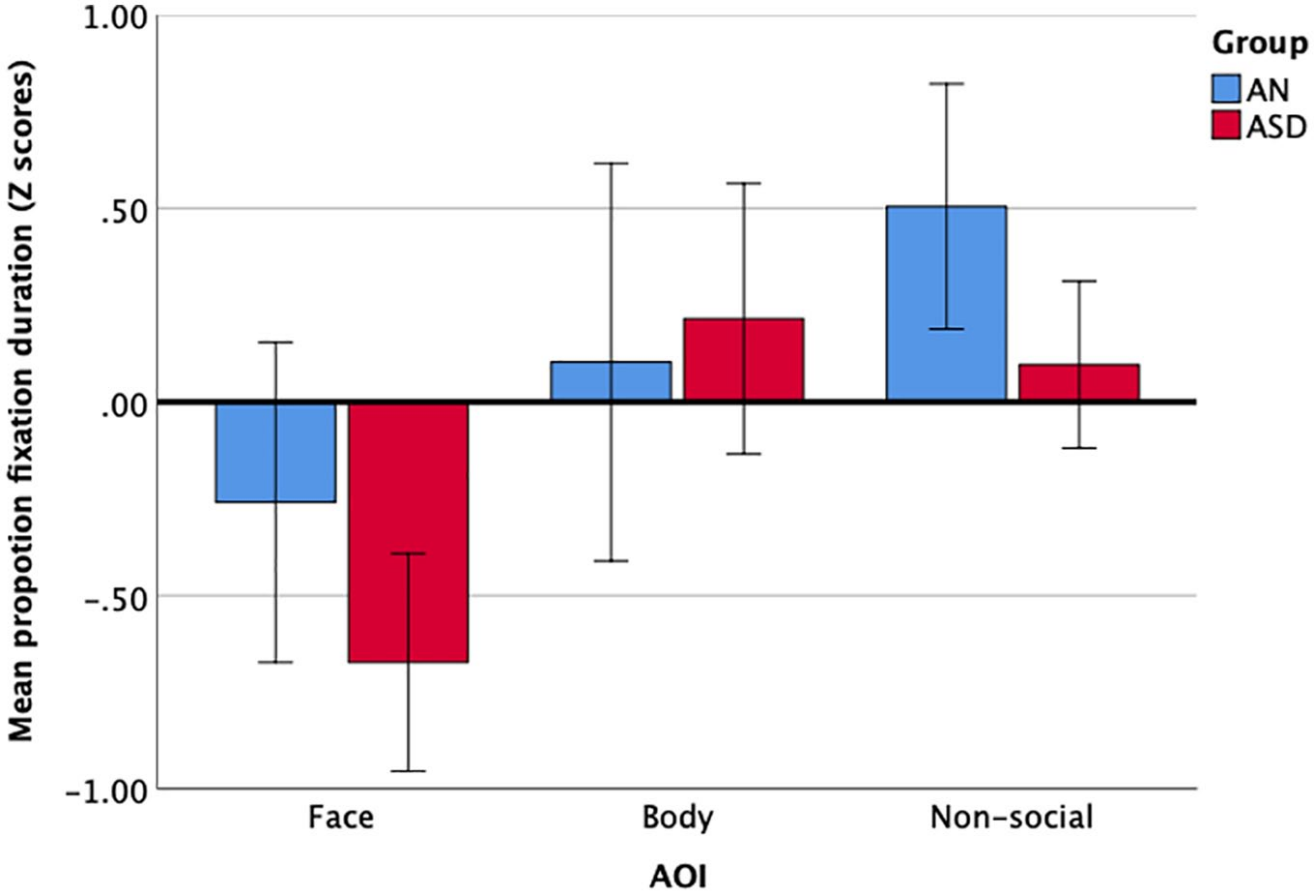
Mean proportion of fixation duration *z*-scores reflecting deviation from sex- and age-matched TD groups. Error bars represent 99% confidence intervals.

To directly compare attention to face, body, and non-social AOIs in individuals with AN and those with ASD, a mixed ANOVA was run using the TD-normed *z*-scores. Mauchley’s test of sphericity was significant (*p* < 0.001), therefore a Greenhouse–Geisser correction was used. There was no significant interaction between AOI and group, *F*(1.74, 233.11) = 1.74, *p* = 0.183, η_*p*_^2^ = 0.01. The main effect of AOI was significant *F*(1.74, 233.11) = 12.66, *p* < 0.001, η_*p*_^2^ = 0.09, indicating that looking times to the face (ASD *M* = −0.67, *SD* = 0.96, AN M = −0.26, *SD* = 1.19) were significantly more atypical than looking times to the body (ASD *M* = 0.22, *SD* = 0.10, AN *M* = 0.10, *SD* = 1.30), *p* = 0.004, or non-social AOI (ASD *M* = 0.10, *SD* = 0.71, AN *M* = 0.50, *SD* = 0.95), *p* < 0.001, relative to controls. The main effect of group was also significant, *F*(1,134) = 17.65, *p* < 0.001, η_*p*_^2^ = 0.12, participants with ASD showed greater differences in looking times from their control group than did the AN group, *p* < 0.001. Visualising the data shows that differences in looking patterns between AN and ASD and their respective control groups were largest for faces, with less attention to this AOI in the clinical groups, as shown in Figure 3. Furthermore, participants with ASD showed more atypical looking duration overall compared with participants with AN, but this did not vary by AOI.

A sensitivity analysis in females only was conducted to examine whether the differing sex ratios in AN and ASD were responsible for differences in attention (see Supplementary File 2). Similar to the full sample *z*-score analysis, there was no interaction between AOI and group, and participants were more atypical in looking times to faces than to the body or non-social AOIs. However, in females the effect of group became non-significant, and the effect size reduced, suggesting deviations from the norm became more similar in females with AN and ASD.

There were no differences in patterns of attention based on psychiatric medication status in the two clinical groups (see Supplementary File 3).

### Associations between social attention and ASD symptoms

No significant correlations were found between the proportion of time spent looking at face, body or non-social AOIs and SRS-2 *t* score subscales, anxiety or depression in AN or ASD (all *p* > 0.01).

## Discussion

The aim of the current study was to examine social attention in individuals with AN and those with ASD compared with sex- and age-matched control groups. Furthermore, we aimed to establish whether social motivation may underlie possible differences in social attention across both disorders. We hypothesised that both individuals with AN and those with ASD would display reduced attention to face AOIs compared with the controls. In partial support of this hypothesis, individuals with ASD showed reduced looking times to faces compared with TD-ASD. However, this pattern was not replicated in AN; no differences in attention to faces or the other AOIs were found when comparing AN and TD-AN. Analyses of *z*-scores indicated that in accordance with our hypothesis, attention to faces was more atypical than attention to body or non-social AOIs across AN and ASD. However, deviations from the norm were greater in ASD than in AN. We also hypothesised that difficulties in social motivation would predict reduced attention to faces in ASD and AN. This hypothesis was not supported; difficulties in social motivation were not associated with attention to face, body, or non-social AOIs.

Previous research has suggested reduced attention to faces and increased attention to non-social information in people with ASD (Frazier et al., 2017). This pattern of attention is reported across the lifespan and has been proposed as an early marker of ASD (Jones et al., 2017). In accordance with these findings, our study demonstrated reduced attention to faces in adults with ASD. While TD participants looked at faces and non-social aspects of the scene for similar proportions of time, participants with ASD preferred to look at non-social areas more than faces. Reduced attention to faces from infancy may result in reduced social-cognitive learning opportunities, and contribute to social skill and communication impairments in people ASD (Del Bianco et al., 2020; Gui et al., 2021). However, our results did not suggest that difficulties in social motivation were responsible for or related to reduced attention to faces in people with ASD or AN. There are several reasons for this unexpected finding. First, it could be that social motivation is inadequately captured by the SRS-2. For example, some have suggested that the social motivation subscale measures behavioural approach and maintenance, and therefore may misattribute behavioural avoidance as a lack of social motivation (Elias & White, 2020). Indeed, some studies using the SRS-2 have failed to distinguish between individuals with ASD and those with social anxiety disorder, a disorder characterised by high levels of social avoidance, but not necessarily low social motivation (Capriola-Hall et al., 2021; Cholemkery et al., 2014; South et al., 2017). Individuals with AN also show high levels of social anxiety and avoidance (Courty et al., 2015; Kerr-Gaffney et al., 2018), which may have contributed to their high scores on the social motivation subscale in the present study.

Relatedly, some evidence suggests that instead of reflecting a lack of social motivation, reduced attention to faces may reflect attempts to reduce overstimulation, stress or manage cognitive load in individuals with ASD (Jaswal & Akhtar, 2018). For example, in infant–caregiver interactions, overstimulation in infants is followed by looking away, and a subsequent decrease in heart rate (Field, 1981). Similarly, studies in people with ASD have demonstrated higher levels of autonomic arousal (measured using skin conductance) when viewing faces, compared with TD (Joseph et al., 2008). Maintaining mutual gaze uses cognitive processing resources, therefore looking away from a face or eyes can reduce cognitive load (Phelps et al., 2006). This tactic may be especially useful for people with autism, who may find social information particularly cognitively demanding (Doherty-Sneddon et al., 2012). Qualitative accounts support these findings; some individuals with ASD report that by avoiding looking at their conversational partner, they can listen better to what they are saying (Robledo et al., 2012). Providing evidence against the social motivation hypothesis of autism, qualitative research has also demonstrated that people with autism do desire and gain pleasure from social relationships (Bargiela et al., 2016; Crompton et al., 2020), and experience loneliness to the same degree or more so than TD (Bauminger et al., 2003; Lasgaard et al., 2010). However, due to other characteristics of autism (e.g. sensory sensitivities) as well as social rejection experiences during formative years, individuals with autism may find social interactions extremely stressful (Tierney et al., 2016). Thus, reductions in social attention may act as a means of reducing anxiety. Future research combining eye tracking with autonomic measures during social tasks may be useful in exploring this issue.

Contrary to our hypothesis, participants with AN did not show reduced attention to face AOIs compared with TD. This finding also contrasts with previous research examining attention to faces in AN (Fujiwara et al., 2017; Kerr-Gaffney, Mason, et al., 2021; Watson et al., 2010). It may be that differences in stimuli used across studies are responsible for the mixed results. The affective content of the scene and the social or dyadic nature of presentation are both likely to affect attention, especially in dynamic stimuli were these qualities may vary temporally (Tenenbaum et al., 2021). Past research in AN has mostly used static stimuli, often faces presented in isolation (Dinkler et al., 2019; Fujiwara et al., 2017; Pinhas et al., 2014; Watson et al., 2010). By contrast, the video shown in our study depicted an emotive and highly social scene, in which the protagonist faces social ostracisation while trying to find somewhere to sit in the school cafeteria. It may be that individuals with AN are motivated to attend to relevant parts of the scene when there is a complex social story playing out, but are less attentive when passively looking at images where there is no interaction or ToM required. Indeed, attention to faces has been shown to be task dependent in individuals with autism. Mouga et al. (2021) demonstrated that attention allocation in people with ASD became normalised when they had to narrate what was happening in a story book, as opposed to a simple description of a picture.

Another explanation for the lack of group differences in social attention in AN and TD relates to the sex ratio associated with AN. While AN is far more common in females, with a female to male sex ratio of 4:1 (Bulik et al., 2006), ASD is more common in males (Loomes et al., 2017). In typical development, girls show more attention to faces compared with boys, a pattern that continues throughout adulthood (Gluckman & Johnson, 2013). Furthermore, studies examining sex differences in social attention in ASD often do not find decreased looking times to social stimuli in females with ASD (Harrop et al., 2018, 2020). If female sex acts as a protective factor against social attention difficulties in both the general population and autistic individuals, one might expect a similar effect in AN. If this is the case, the dramatic female sex ratio associated with AN may mean that most individuals with AN do not show difficulties in social attention. Despite the lack of group differences in social attention between AN and AN-TD, our AN and ASD comparisons showed similar patterns of attention allocation across faces, bodies, and non-social regions in AN and ASD. These results suggest that compared with their unaffected control populations, both AN and ASD look less at face AOIs than expected. However, deviation from typical looking patterns across all AOIs were larger in ASD than in AN. Given the sex differences between the two disorders, we also conducted a sensitivity analysis restricting the data to females only. Comparison of *z*-scores in AN and ASD indicated that the differences seen between the two clinical groups were indeed partly due to sex: the effect of group became non-significant, and the effect size reduced. This finding suggests that the differences seen between AN and ASD are partly due to differences in the sex ratio between the two disorders.

### Limitations

The study has several limitations. To measure attention, we used the proportion of time spent looking at each AOI. While this metric is used most often in social attention studies, it does not take temporal variations in attention into account. As mentioned previously, variations in the visual scene, emotional content and social content are likely to affect attention throughout the duration of the clip. Our approach may have obscured any subtle changes in social attention over time, such as disengagement from stimuli. Only a handful of studies have examined temporal variations in social attention in ASD, for example, Del Bianco et al. (2020) demonstrated that both ASD and TD participants showed an initial interest in faces, followed by a decline over the next several seconds. However, TD participants were more likely to return to faces, whereas the probability of participants with ASD looking at faces in latter parts of the trial remained low. Future studies in AN would benefit from examining temporal variations in social attention, as these may explain the mixed findings thus far.

Another limitation relates to potential comorbid diagnoses of participants. We were unable to ascertain whether any of the participants with ASD included in the study had a comorbid eating disorder diagnosis, thus there may have been some diagnostic overlap between the ASD and the AN group. Similarly, although we did have information on comorbid ASD diagnoses in the AN group (no cases of ASD), the original study did not provide a full diagnostic assessment for ASD for participants with AN. Research suggests that around 10% of individuals with AN meet full diagnostic criteria for ASD, however, many do not receive a diagnosis until adulthood (Kinnaird et al., 2019; Westwood et al., 2018). Therefore, there may have been undiagnosed cases of ASD in the AN group. As a result, scores on the SRS-2 and attention characteristics could appear more similar across the clinical groups than they would if there was no diagnostic crossover. Finally, we only included participants with average or above average IQ, as IQs outside this range are rare in individuals with AN (Lopez et al., 2010). Our results are therefore not generalisable to individuals with ASD and intellectual impairment.

## Conclusion

Our results do not support the hypothesis that differences in social motivation underlie reduced social attention in both AN and ASD. Although similar patterns of attention were observed in AN and ASD, that is, reduced attention to faces compared with sex- and age-matched TD models, differences from the TD population were larger in ASD and direct comparison of AN and TD-AN were not significant. Recent work has shown differences in the male and female presentation of ASD, for example, women with ASD show fewer restricted interests and repetitive behaviours, but show more sensory processing abnormalities than males with ASD (Lai et al., 2011; Wilson et al., 2016). Given that AN mostly affects females, future studies directly comparing females with AN and females with ASD are required to understand which traits may be responsible for the common behavioural difficulties seen in both disorders. Furthermore, longitudinal studies are required to understand whether differences in social attention in AN vary with illness severity, or whether they represent a stable trait as seen in ASD.

## Supplemental Material

sj-docx-1-aut-10.1177_13623613211060593 – Supplemental material for Social attention in anorexia nervosa and autism spectrum disorder: Role of social motivation Click here for additional data file. Supplemental material, sj-docx-1-aut-10.1177_13623613211060593 for Social attention in anorexia nervosa and autism spectrum disorder: Role of social motivation by Jess Kerr-Gaffney, Emily Jones, Luke Mason, Hannah Hayward, Declan Murphy, Eva Loth and Kate Tchanturia in Autism

sj-docx-2-aut-10.1177_13623613211060593 – Supplemental material for Social attention in anorexia nervosa and autism spectrum disorder: Role of social motivation Click here for additional data file. Supplemental material, sj-docx-2-aut-10.1177_13623613211060593 for Social attention in anorexia nervosa and autism spectrum disorder: Role of social motivation by Jess Kerr-Gaffney, Emily Jones, Luke Mason, Hannah Hayward, Declan Murphy, Eva Loth and Kate Tchanturia in Autism

sj-docx-3-aut-10.1177_13623613211060593 – Supplemental material for Social attention in anorexia nervosa and autism spectrum disorder: Role of social motivation Click here for additional data file. Supplemental material, sj-docx-3-aut-10.1177_13623613211060593 for Social attention in anorexia nervosa and autism spectrum disorder: Role of social motivation by Jess Kerr-Gaffney, Emily Jones, Luke Mason, Hannah Hayward, Declan Murphy, Eva Loth and Kate Tchanturia in Autism
