# Supplementary material for: Social attention in anorexia nervosa and autism spectrum disorder: Role of social motivation
Source: Autism. 2021 Nov 30;26(7):1641–55. doi: 10.1177/13623613211060593 (PMC9483678; doi:10.1177/13623613211060593)
Supplement: sj-docx-1-aut-10.1177_13623613211060593 – Supplemental material for Social attention in anorexia nervosa and autism spectrum disorder: Role of social motivation [file sj-docx-1-aut-10.1177_13623613211060593.docx]

**Additional file 1**

**Participant recruitment**

The EU-AIMS Longitudinal European Autism Project is a multi-site study across six European specialist ASD centres: Institute of Psychiatry, Psychology and Neuroscience, King’s College London (IoPPN/KCL, United Kingdom), Autism Research Centre, University of Cambridge (UCAM, United Kingdom), University Medical Centre Utrecht (UMCU, Netherlands), Radboud University Nijmegen Medical Centre (RUNMC, Netherlands), Central Institute of Mental Health (CIMH, Germany) and the University Campus Bio-Medico (UCBM) in Rome, Italy. Participants with ASD and TD-ASD were recruited from a variety of sources including existing volunteer databases, existing research cohorts, clinical referrals from local outpatient centres, special needs schools, mainstream schools and local communities. Baseline recruitment took place between January 2014 and March 2017.

Participants with AN and TD-AN took part in a wider study investigating social and emotional functioning in AN. The study took place at the IoPPN KCL. The study was cross-sectional and recruitment took place between February 2018 and June 2019. Participants were recruited through KCL staff and student notices, advertisements in the local community, and online (B-eat, call for participants, and MQ mental health). Participants with AN were also recruited from two NHS specialist eating disorder services: South London and Maudsley trust and Central and North West London trust.
