# Supplementary material for: Social attention in anorexia nervosa and autism spectrum disorder: Role of social motivation
Source: Autism. 2021 Nov 30;26(7):1641–55. doi: 10.1177/13623613211060593 (PMC9483678; doi:10.1177/13623613211060593)
Supplement: sj-docx-2-aut-10.1177_13623613211060593 – Supplemental material for Social attention in anorexia nervosa and autism spectrum disorder: Role of social motivation [file sj-docx-2-aut-10.1177_13623613211060593.docx]

**Additional file 2**

**Sensitivity analysis – females only**

A mixed ANOVA was run to compare ASD and AN in the proportion of time spent looking at each AOI (face, body, non-social). There was no significant interaction between AOI and group, F(2, 128) = 1.45, p = .238, ηp^2^ = 0.02. There was a significant effect of AOI, F(2, 128) = 7.94, p = .001, ηp^2^ = 0.11. Participants were significantly more atypical in looking times to the face (ASD M = -0.62, SD = 0.86; AN M = -0.30, SD = 1.24) than to the body (ASD M = 0.42, SD = 1.03; AN M = 0.19, SD = 1.31), p = .010, and non-social AOIs (ASD M 0.06, SD = 0.72, AN M = 0.54, SD = 0.97), p = .002. There was no significant effect of group on looking times, F(1, 64) = 5.66, p = .020, ηp^2^ = .08, indicating that deviations from the control groups were of similar magnitude in AN and ASD.
