# Supplementary material for: Social attention in anorexia nervosa and autism spectrum disorder: Role of social motivation
Source: Autism. 2021 Nov 30;26(7):1641–55. doi: 10.1177/13623613211060593 (PMC9483678; doi:10.1177/13623613211060593)
Supplement: sj-docx-3-aut-10.1177_13623613211060593 – Supplemental material for Social attention in anorexia nervosa and autism spectrum disorder: Role of social motivation [file sj-docx-3-aut-10.1177_13623613211060593.docx]

**Additional file 3**

**Medication status and attention**

A mixed ANOVA was performed comparing participants with AN who were taking any type of psychiatric medication (n = 22) to those who were not (n = 21). Mauchley’s test of sphericity was significant (p <.001), therefore a Greenhouse-Geisser correction was used. There was no significant interaction between AOI and medication status, F(1.50, 61.44) = 0.42, p = .600, ηp^2^ = 0.01. The main effect of AOI was significant, F(1.50, 61.44) = 78.87, p <.001, ηp^2^ = 0.66, participants looked at faces and non-social aspects of the scene more than bodies, both p<.001.

A mixed ANOVA was performed comparing participants with ASD who were taking any type of psychiatric medication (n = 27) to those who were not (n = 66). Mauchley’s test of sphericity was significant (p <.001), therefore a Greenhouse-Geisser correction was used. There was no significant interaction between AOI and medication status, F(1.67, 140.09) = 0.00, p = .992, ηp^2^ = 0.00. The main effect of AOI was significant, F(1.67, 140.09) = 195.06, p <.001, ηp^2^ = 0.70, participants looked at non-social AOIs more than faces and bodies, and faces more than bodies (all p<.001).
